# Retraction Note: Increased survival and cell cycle progression pathways are required for EWS/FLI1-induced malignant transformation

**DOI:** 10.1038/s41419-019-1853-1

**Published:** 2019-08-13

**Authors:** Tahereh Javaheri, Zahra Kazemi, Jan Pencik, Ha T. T. Pham, Maximilian Kauer, Rahil Noorizadeh, Barbara Sax, Harini Nivarthi, Michaela Schlederer, Barbara Maurer, Maximillian Hofbauer, Dave N. T. Aryee, Marc Wiedner, Eleni M. Tomazou, Malcolm Logan, Christine Hartmann, Jan P. Tuckermann, Lukas Kenner, Mario Mikula, Helmut Dolznig, Aykut Üren, Günther H. Richter, Florian Grebien, Heinrich Kovar, Richard Moriggl

**Affiliations:** 1 Ludwig Boltzmann Institute for Cancer Research, Vienna, Austria; 2 Institute of Animal Breeding and Genetics, University of Veterinary Medicine, Vienna, Austria; 3 Medical University of Vienna, Vienna, Austria; 4 Center of Physiology and Pharmacology, Vienna, Austria; 5 Clinical Institute of Pathology, Vienna, Austria; 6 Children’s Cancer Research Institute, St. Anna Kinderkrebsforschung, Vienna, Austria; 7 CeMM Research Center for Molecular Medicine of the Austrian Academy of Sciences, Vienna, Austria; 8 Department of Pediatrics, Medical University of Vienna, Vienna, Austria; 9 Randall Division of Cell and Molecular Biophysics, King’s College London, London, UK; 10 Department of Bone and Skeletal Research, Institute of Experimental Musculoskeletal Medicine, University Hospital Münster, Münster, Germany; 11 Institute of General Zoology and Endocrinology, University of Ulm, Ulm, Germany; 12 University of Veterinary Medicine, Vienna, Austria; 13 Institute of Medical Genetics, Vienna, Austria; 14 Georgetown University Medical Center, Lombardi Comprehensive Cancer Center, Washington, USA; 15 Children’s Cancer Research Centre and Department of Pediatrics, Klinikum rechts der Isar, Technische Universität München, Munich, Germany

**Retraction Note to: Cell Death & Disease**

10.1038/cddis.2016.268

Published online 13 Oct 2016

The authors have retracted this article because in Supplementary Figure 8b (1) the left-hand and right-hand MCL1 blots are the same, and (2) the cleaved caspase-3 blot lane 5 in the left panel is a duplicate of the first lane of the cleaved caspase-3 blot in the middle panel. Authors M. Hofbauer, M. Wiedner, C. Hartman, J. Tuckerman, J. Pencik, E. Tomazou, M. Schlederer, T. Javaheri, M. Mikula, H. Nivarthi, L. Kenner, D. Aryee, B. Sax, F. Grebien, H. Dolznig, G. Richter, M. Logan, B. Maurer, A. Üren, H. Kovar, R. Morrigl, and H. Pham agree with the retraction. Authors Z. Kazemi, M. Kauer, and R. Noorizadeth have not responded to correspondence about this retraction. The authors are repeating their experiments and a new manuscript will be submitted for peer review.

